# A Finite Element Study to Investigate the Mechanical Behaviour of Unidirectional Recycled Carbon Fibre/Glass Fibre–Reinforced Epoxy Composites

**DOI:** 10.3390/polym13183192

**Published:** 2021-09-21

**Authors:** Sankar Karuppannan Gopalraj, Timo Kärki

**Affiliations:** Fiber Composite Laboratory, Department of Mechanical Engineering, LUT University, P.O. Box 20, 53850 Lappeenranta, Finland; timo.karki@lut.fi

**Keywords:** finite element methods, recycled composites, carbon fibre, glass fibre, elastoplastic material, ductile damage

## Abstract

Recycled carbon fibre–reinforced epoxy (rCF/EP) composites and recycled glass fibre–reinforced epoxy (rGF/EP) composites were numerically investigated to examine their mechanical properties, such as uniaxial tensile and impact resistance, using finite element (FE) methods. The recycled composites possess unidirectional, long and continuous fibre arrangements. A commercially available Abaqus/CAE software was used to perform an explicit non-linear analysis with a macroscale modelling approach, assuming the recycled composites as both homogenous and isotropic hardening. Five composite types were subjected to a numerical study based on the recycled fibre’s volume fraction (40 and 60%) of rCF/EP and rGF/EP, along with (100%) fibreless cured epoxy samples. The materials were defined as elastoplastic with a continuum ductile damage (DUCTCRT) model. The experimental tensile test results were processed and calibrated as primary input data for the developed FE models. The numerical tensile results, maximum principal stress and logarithmic strain were validated with their respective experimental results. The stress–strain curves of both results possess a high accuracy, supporting the developed FE model. The numerical impact tests examined the von Mises stress distribution and found an exponential decrease in the stiffness of the composite types as their strength decreased, with the 60% rCF/EP sample being the stiffest. The model was sensitive to the mesh size, hammer velocity and simulation time step. Additionally, the total internal energy and plastic dissipation energy were measured, but were higher than the experimentally measured energies, as the FE models eliminated the defects from the recycled process, such as a poor fibre wettability to resin, fibre bundle formation in rCFs and char formation in rGFs. Overall, the developed FE models predicted the results for a defect-free rCF/EP and rGF/EP composite. Hence, the adopted modelling techniques can validate the experimental results of recycled composites with complex mechanical properties and damage behaviours in tensile and impact loading conditions.

## 1. Introduction

Recent progress in recycling methods, such as thermal, chemical and solvolysis using water and mild solvents to recycle carbon fibre-reinforced polymer (CFRP) composite wastes and glass fibre-reinforced polymer (GFRP) composite wastes, have established a new era towards sustainable waste disposal. In particular, advanced recycling techniques, namely closed-loop and open-loop recycling, managed to reuse the recycled carbon fibres (rCFs) and recycled glass fibres (rGFs) repeatedly into either their identical or modified applications in order to close their life cycle loop, encouraging a circular economy [1]. The rCFs and rGFs possess mechanical properties similar to their virgin counterparts. In addition, only one-third of energy (in some cases, even less) is required for recycling composite wastes compared to manufacturing virgin fibres and composites. Hence, CFRP and GFRP composite wastes have become valuable raw materials for recycling industries. As a result, laboratory-scale recycling processes with a higher fibre yield and recycling efficiency are transforming into industrial-scale recycling processes to recycle and reuse fibres in enormous quantities to satisfy the global demand, replacing virgin composites [1,2,3,4,5,6,7].

Further extensive investigations into the mechanical properties are required at the micro and macro levels to implement the recycled composites into an actual application, such as automobiles, aeronautics, windmills and other high-performance fields. Typical studies observed from the literature [1,2] primarily focus on testing recycled fibres without a matrix and with the matrix as fibre-reinforced polymer (FRP) composites in order to analyse the overall mechanical properties and failure modes in various testing conditions, such as tensile, compression, shear and impact.

Notable studies in recycled (r) rCFRP, such as Pimenta et al. 2010 [8], studied the mechanical properties and fracture behaviour of rCFRP composites to optimise the recycling process and develop the recycled composite design. Later, Pimenta and Pinho 2012 [9] briefly studied rCFs at both micro (as fibres) and meso-level (as composites) in tensile, compression and shear loading conditions, and compared the properties to their virgin form. It concluded that the stiffness of the recycled composites remains the same despite different loading conditions. Furthermore, in 2014, Pimenta and Pinho [10] studied rCFRP composites, highlighting the fibre bundle (defects from the recycling process), as it toughens the composites without reducing their stiffness or strength. The study also investigated the rCFRP at micro, meso and macro-level. Similar studies in rGFRP composites, such as Feih et al. 2011 [11], studied thermally recycled GFRP at micro and meso-scale. The study investigated the tensile strength reduction in the rGFRP due to the recycling temperature. Yang et al. 2015 [12] also studied rGFRP at micro and meso-scale to recover the strength of the rGFs using chemical treatments in tensile, flexural and impact modes. The study successfully regenerated the rGFs strength to achieve closed-loop recycling. However, such existing studies, observed from the literature, focused purely on experimental investigations. To understand the overall mechanical and fracture behaviours of the FRP composite, predominantly tensile and impact, it is required to investigate experimental testing along with numerical analysis [13,14,15,16,17].

Research involving finite element (FE) methods to analyse virgin (v) vCFRP and vGFRP composites have been well developed and established. Several studies have experimentally and numerically investigated the (tensile and impact) mechanical properties and failure behaviours. These can be found in literature reviews, such as Laffan et al. 2012 [18], Wang and Huang 2018 [19], Fatima et al. 2019 [20] and Müzel et al. 2020 [21]. However, these studies purely focused on virgin composites and recorded no significant studies involving recycled composites. Therefore, theories from virgin composite studies have to be analysed and incorporated to perform such studies over recycled composites. Based on the previous studies involving FE methods for FRP composites, both the elastoplastic material behaviour for composite modelling and a ductile damage model for fracture behaviours seem promising.

These numerical approaches were widely used for virgin FRP composites and can be observed in various stages of evolution in previous literature, such as Ju and Lee 2001 [22], who studied an unidirectional (UD) FRP composite’s ductile behaviour in the matrix using an elastoplastic-based damage model and predicted the damage mechanism. The numerically predicted stress–strain (SS) behaviours have higher-order similarities compared with their experimental results. González et al. 2004 [23] studied composites using homogenised models, adopting elastoplastic material behaviour to investigate the tensile and shear properties of the composites. The study used experimental data as inputs for numerical analysis to define the non-linear behaviours at the onset of plastic deformation. Totry et al. 2010 [24] studied the in-plane shear response of the CFRP composites, considering the non-linear composite behaviour as elastoplastic behaviour, and validated the numerical model using experimental data. Melro et al. 2013 [25,26] studied UD continuous fibre-reinforced composites, similar to the materials used in this study, but in virgin form. Part I [25] studied the micro-scale material behaviour as elastoplastic with an isotropic damage constitutive model to investigate the composite failure behaviours. Part II [26] studied damage initiation and propagation in various loading conditions, such as transverse tension, compression, shear and longitudinal shear. 

Further developments in adopting similar numerical modelling approaches can be seen in the latest studies, such as Ghayoor et al. 2019 [27] and Ahmadian et al. 2020 [28], who have investigated UD CFRP composite damage behaviours, highlighting resin-rich zones in the matrix (epoxy) using elastoplastic material behaviour. Ghayoor et al. 2019 [27] studied the effects of failure initiation at the resin-rich areas. The study concluded that composites fail at lower strain rates, as the failure initiation occurs at the fibre clusters. Ahmadian et al. 2020 [28] developed the study further by using a ductile damage model to study the composite failure development in tension, compression and shear modes. The study concluded that the resin-rich areas are susceptible to nucleation under tension loading, resulting in an overall strength reduction and failure. Liu et al. 2020 [14] used an elastoplastic damage model to predict the impact behaviour of the UD CFRP composites and validated the models using experimental impact test results. The study successfully managed to replicate the experimental impact damage behaviour using FE methods. Yadav and Thapa [29] studied GFRP and developed a strain-based continuum damage model to study the decreasing elasticity and stiffness degradation under fatigue damage, and validated the results. Khosravani and Zolfagharian [30] examined the tensile behaviours of fibreless polymers showcasing elastoplastic behaviour using experimental methods, and validated the non-linear behaviours using ductile failure models.

Based on the literature studies, a research gap appears, where previously no significant studies have numerically investigated the mechanical properties, especially the uniaxial tensile and impact resistance behaviours of UD and continuous and long rCFRP and rGFRP composites, and numerically predicted their damage behaviours by applying FE methods. As recycling technologies advance, and more rCFs and rGFs are available to replace virgin fibres, there is a need for such a study to explore these complex mechanical properties and failure behaviours by combining both experimental and numerical methods.

This study aims to numerically investigate the mechanical properties and damage behaviour of recycled carbon fibre–reinforced epoxy (rCF/EP) and recycled carbon fibre–reinforced epoxy (rGF/EP) composites, along with cured laminating epoxy (EP) materials, based on a macro-mechanical approach, embracing FE theories from virgin composite studies. Explicit non-linear analyses are performed to validate two standardised test methods: the tensile test (TT) and impact test (IT). The material modellings to map the non-linear behaviours are performed assuming the rCF/EP, rGF/EP and EP materials as elastoplastic. For damage, a continuum ductile damage model is used to predict their damage behaviours in uniaxial tensile and impact modes. The experimental TT results from the previous study [31] are processed as primary input data for all of the numerical test models. Finally, the numerical results from the TT and IT are validated and discussed for their respective experimental results. Hence, the study will provide insights into experimental—data processing and verification, numerical—material modelling, tensile damage prediction and the impact resistance behaviour of the rCF/EP and rGF/EP composites.

This paper is organised initially by describing the used composite types and their mechanical properties and damage behaviours during experimental testing in Section 2. Section 3 consists of the overall adopted FE methodology. Initially, a general composite behaviour is assumed. Then, equations defining the chosen material and damage modelling are presented. Subsequently, experimental data selection, calibration and processing as an input for numerical analysis are presented. Finally, FE—modelling, loads and boundary conditions are presented. Section 4 describes the calculated numerical input data and their results after numerical TT and IT. In addition, discussions involving experimental vs. numerical values, along with IT predictions, are presented. After the Results and Discussion, the paper ends with a Conclusion in Section 5.

## 2. Materials and Their Behaviour

### 2.1. Recycled Composites

The materials used in this research work were taken from the author’s previous study [31]. It comprises of rCF/EP, rGF/EP and EP composite without fibres. These composites were obtained as a result of recycling CFRP and GFRP composite wastes containing valuable carbon and glass fibres using a novel thermal recycling process. The recycled fibres (rCFs and rGFs) were subsequently compression moulded using fresh EP. The newly produced rCF/EP and rGF/EP composites, along with the EP samples, were mechanically tested to measure both their uniaxial tensile properties using ISO 527-2 standard [32] and their impact resistance behaviour using unnotched charpy impact ISO 179-1 standard [33]. Table 1 presents the experimentally measured TT and IT results of the new composites. These values are used as primary data in this current study. As seen, the rCF/EP and rGF/EP composites consist of two types, based on the fibre weight fraction (V^f^) 40 wt% and 60 wt%. The resin combined with its hardener in a 2:1 ratio occupies the composite’s remaining volume (V^r^). The recycled composites possess a uniform structure throughout the lamina. The composite types maintained a UD fibre orientation (0°) and continuous (uniform length from end to end) and long (105 ± 2 mm) fibres.

### 2.2. Recycled Composite Damage Behaviour

**Figure 1:**
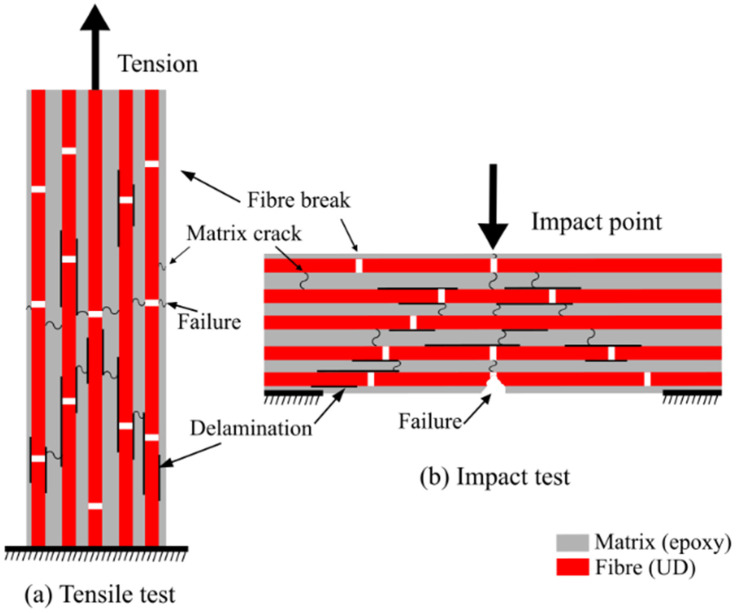
Damage sequence of UD FRP composites tensile mode (modified from [35]) and impact mode (modified from [39]).

The recycled composites damage behaviours are dynamic and heavily influenced by their recycling and remanufacturing processes. To understand such complex behaviours, it is required to compare them with well established and studied damage behaviours of virgin composites. Typically, in virgin UD FRP composites, the failure depends on the fibre, matrix and fibre–matrix interface [24], as all three contribute to the failure initiation and development. In particular, the matrix possesses a significant role in composite damaging, as the matrix strain to failure dominates the fibre strain. The fibre arrangement and direction and the loading conditions (tensile, compression, shear and impact) significantly influence the overall composite damage behaviour [34]. In this study, tensile and impact loading modes were taken for the investigation. Under uniaxial tension loading (see Figure 1), the UD fibres encounter multiple interlaminar breakages at random spots, decreasing the overall composite stiffness. Subsequently, debonding occurs at the fibre–matrix interface, leading to interlaminar composite delamination. As the tension develops, the stress propagates into the matrix, causing crack growth near the broken fibres and leading to a final fracture [35,36]. Similar damage behaviours were observed during the experimental testing [31] for the rCF/EP and rGF/EP composite samples.

Under unnotched charpy impact testing (see Figure 1), especially low-velocity impact, UD FRP composites experience plastic deformations, leading to the matrix cracking followed by a series of failures, such as fibre breakage and delamination. In an IT, composite failure initiation and development occur opposite (tension zone) at the impactor and samples contact point (compression zone). The fracture develops towards the impactor [14,17,20,37]. The UD FRP composites possess a low interlaminar fracture toughness due to their fibre arrangement, and, under impact loading, delamination occurs favourably, resulting in higher impact damage [38]. Similar failure behaviours were observed during experimental impact testing [31] rCF/EP and rGF/EP composite samples. However, the EP samples displayed an explosive impact fracture, as the samples are fibreless.

**Figure 2:**
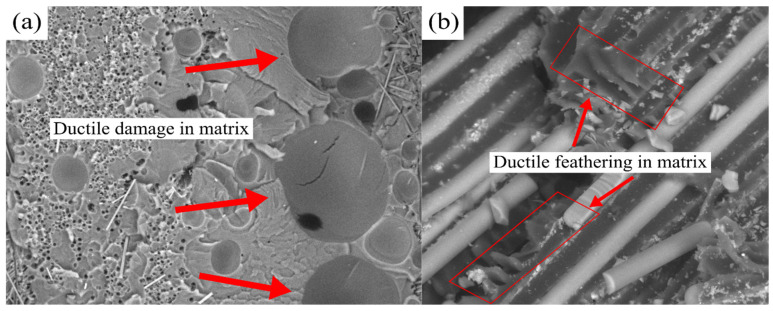
The evidence for ductile damages in matrix: (**a**) rCF/EP; (**b**) rGF/EP.

Figure 2 presents the microscopic images of the rCF/EP and rGF/EP samples from their fractured region after experimental testing from the author’s previous study [31]. These images were taken using a Jeol JSM-5800 LV scanning microscope at 4k magnification. As observed during the testing and microscopic images, most of the rCF/EP and rGF/EP samples failed normal to the fibre directions due to fibre damage, delamination, matrix cracking, fibre pullouts, internal matrix voids and, finally, matrix shear bands in resin-rich zones. Overall, the samples showed multiple matrix-dominating failures. In addition, evidence for ductile behaviour was noticed in the epoxy matrix failure zones. As seen in Figure 2a, ductile-damage-based void nucleation and growth were observed at the resin-rich matrix zones. In addition, in Figure 2b, ductile feathering was observed in multiple spots at the matrix.

**Figure 3:**
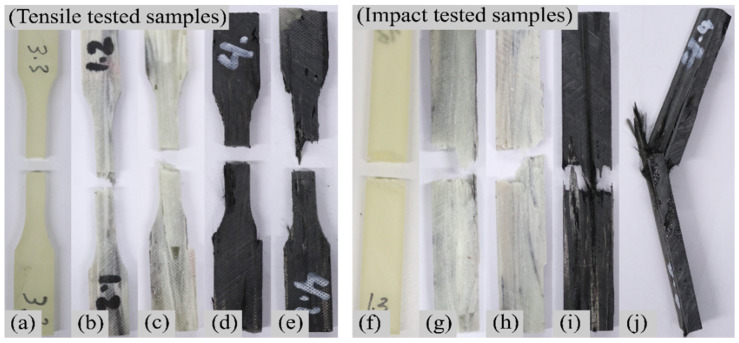
Sample fracture after experimental TT: (**a**) 100% EP; (**b**) 40% rGF/EP; (**c**) 60% rGF/EP; (**d**) 40% rCF/EP; (**e**) 60% rCF/EP; and after IT: (**f**) E-EP; (**g**) 40% rGF/EP; (**h**) 60% rGF/EP; (**i**) 40% rCF/EP; (**j**) 60% rCF/EP.

Figure 3 presents the fractured sample’s images after experimental tensile and impact testing from the author’s previous study [31]. Figure 3a–e presents the images after TT and Figure 3f–j after IT. The images show that the tensile-tested samples broke without any neck formation, indicating a brittle failure. However, the SS values of the tested composites displayed a small elastic zone and a large non-elastic zone with failure initiation and evolution after the peak values, indicating ductile damage domination. Apart from the regular damage behaviours, the composites were influenced by external factors. In rCF/EP composite types, mostly in 60% rCF/EP, the poor wettability (defects from thermal recycling [31]) of the rCFs with the matrix created a weak interfacial strength and influenced the rCF/EP tensile and impact failure.

In UD FRPs, when the fibre properties are superior to the matrix, it creates a weak fracturable nature when aligned as normal to the fibre direction. It favours the crack distribution parallel to the fibre direction [40]. Furthermore, the stress concentrations are weak on the regions of unwet fibre clusters, promoting damage initiation and development towards the matrix zones [41]. Hence, it is known that the inter-fibre distance between each fibre has a heavy influence on the sample damaging [42]. Due to unwet rCFs merging into clusters, some samples developed resin-rich zones that influenced the crack propagation parallel to the fibre direction. Such poorly wet fibres were absent in rGF/EP types. However, the presence of char (resin residue from the recycling process [31]) caused a negative influence on the sample damage. The EP samples under TT and IT showed uniform results throughout the sample population.

## 3. Finite Element Methodology

**Figure 4:**
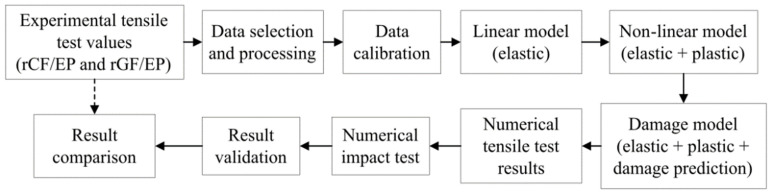
Implemented FE approach for the recycled composites.

Figure 4 presents the overall methodology adopted. Commercially available FE software Abaqus/CAE was used to perform both the numerical TTs and ITs. The tensile and impact models were developed based on explicit non-linear analysis using the experimentally measured TT data as inputs. The FE analysis was performed for all five composite types: EP (100%), rGF/EP (40 and 60%) and rCF/EP (40 and 60%).

### 3.1. Recycled Composite Assumption

In general, rCF/EP and rGF/EP composites possess complex mechanical behaviour compared to vCF/EP and vGF/EP composites. Both the recycling and remanufacturing processes significantly influence their mechanical properties, making it challenging to compare standard composite behaviours. In this study, a UD (0°) and continuous (end-to-end) and long (105 ± 2 mm) fibre arrangement was constantly maintained for the rCF/EP and rGF/EP composite types. Similar structured vCFRP composites lamina are transversely isotropic [15,43,44], or, in some cases, even anisotropic [21]. The vGFRP and EP behave as elastic isotropic solids [36], even though vGFRP microscopically behaves as transversely isotropic [13]. In general, composite assumptions were made depending on the adopted methodologies.

The UD FRP composites are homogenous along their fibre direction. In this study, the experimental uniaxial TT provided results for the composites concerning a single axis (tensile). However, the recycled composite’s compression, shear and individual fibre (rCFs and rGFs) elasticity properties are unknown due to the limitations in handling the recycled fibres. Overall, a macroscale numerical modelling was performed considering all the composite types—EP, (40% and 60%) rGF/EP and (40% and 60%) rCF/EP—as homogenous and isotropic hardening to avoid complexity by empirically assuming the data and fully utilising experimentally measured values.

### 3.2. Elastoplastic Material Model for Recycled Composites

The experimental TT results (SS values) of EP, rGF/EP and rCF/EP composite types initially recorded a smaller linear elastic region. Furthermore, with the applied force (tension), the region developed into a larger non-linear inelastic region. Finally, the SS curves decreased and stopped after reaching their respective ultimate points, indicating the sample fracture. The elastic region (fibre behaviour) can be defined by the Young modulus and Poisson ratio in UD FRP composites. The non-linear region is described using Hahn and Tsai’s [45] equation. However, due to the observed recycled composite behaviour, the non-linear response of the samples can also be explained by defining the composite matrix as elastoplastic behaviour [13]. Such elastoplastic behaviour is commonly observed in UD FRP composites and plays a significant role in predicting composite strength, failure and damage evaluation [19,25,46,47].

Based on overall observations, elastoplastic material modelling was used in this study. The model [48] assumes that the plastic deformation ($F^{\mathrm{pl}}$) follows after elastic deformation ($F^{\mathrm{el}}$) to form an overall deformation ($F^{T}$).

(1) $$F^{T}={\;F}^{\mathrm{el}}+F^{\mathrm{pl}}$$

The deformation identification and separation are based on an additive relationship between the strain rates, where the total strain rate (${\overset{˚}{\varepsilon }}^{T}$) is defined as the sum of elastic (${\overset{˚}{\varepsilon }}^{\mathrm{el}}$) and plastic strain rates (${\overset{˚}{\varepsilon }}^{\mathrm{pl}}$) [48].

(2) $${\overset{˚}{\varepsilon }}^{T}={\overset{˚}{\varepsilon }}^{\mathrm{el}}+{\overset{˚}{\varepsilon }}^{\mathrm{pl}}$$

The damage model involves elastoplastic material behaviour with ductile damage as dominant. Therefore, Equation (3) [28,49] represents the yield surface of the model, in which, the experimental SS curves are used as an input to determine the yield function $\sigma_{Y}({\bar{\varepsilon }}_{\mathrm{eq}}^{\mathrm{pl}})$, where, ${\bar{\varepsilon }}_{\mathrm{eq}}^{\mathrm{pl}}$ = the equivalent plastic strain.

(3) $$f\left(\sigma \right)=q\;-{\;\sigma }_{Y}\left({\bar{\varepsilon }}_{\mathrm{eq}}^{\mathrm{pl}}\right)$$

### 3.3. Continuum Ductile Damage Model for Recycled Composites

As the composite types are defined as elastoplastic behaviour, studies using similar materials [25,26] have proposed isotropic damage models to predict the sample damage evolution. Failure initiation usually occurs in the matrix in UD FRP composites, making the composites ductile-dominating failures [50,51]. Similarly, the experimental TT values highlight a ductile-based behaviour dominating the overall composite outcomes, which is numerically implemented using a continuum ductile damage model. Hooputra et al. 2004 [49] laid the foundation for the ductile-based failure of the cured epoxy matrix (brittle material) under tension loading. The matrix encountered a noticeable plastic deformation before failure initiation and development.

As seen in Figure 2a,b, the void nucleation, growth and coalescence were the foundation for developing and incorporating the damage model. The damage model [48,49] assumes that the onset of damage (${\bar{\varepsilon }}_{D}^{\mathrm{pl}}$) is a function of stress triaxiality ($\eta =-\frac{\sigma_{m}}{\sigma_{\mathrm{eq}}}$) and the equivalent plastic strain rate (${\overset{˚}{\bar{\varepsilon }}}_{0}^{\mathrm{pl}}$), as shown in Equation (4), where $\sigma_{m}$ = stress state hydrostatic component and $\sigma_{\mathrm{eq}}$ = Huber–von Mises equivalent stress.

(4) $${\bar{\varepsilon }}_{D}^{\mathrm{pl}}\left(\eta ,{\overset{˚}{\bar{\varepsilon }}}_{0}^{\mathrm{pl}}\right)$$

The stress triaxiality is further defined in Equation (5) [48], where trace (T) = trace of the stress tensor, which equals the sum of principal stresses. For the performed uniaxial TT, the following stress conditions are considered, $\sigma_{2}{=\sigma }_{3}{=\sigma }_{12}{=\sigma }_{31}{=\sigma }_{23}=0$. Finally, the stress triaxiality for uniaxial η = −0.333 and the equivalent plastic strain rate ${\overset{˚}{\bar{\varepsilon }}}_{0}^{\mathrm{pl}}$ = 0, as the materials are strain-rate-independent.

(5) $$\eta =-\frac{\frac{1}{3}\times \mathrm{trace}\;\left(T\right)}{\sigma_{\mathrm{mises}}}=-\frac{\frac{1}{3}\times \left(\sigma_{\mathrm{xx}}+\sigma_{\mathrm{yy}}+\sigma_{\mathrm{zz}}\right)}{\sigma_{\mathrm{xx}}}$$

For the damage to initiate, the equivalent plastic strain (${\bar{\varepsilon }}_{\mathrm{eq}}^{\mathrm{pl}}$) reaches a threshold value known as fracture strain (${\bar{\varepsilon }}_{0}^{\mathrm{pl}}$), where the variable D = 0, in which, the plastic deformation increases monotonically as the state variable ($\omega_{D}$) increases. The damage initiation occurs when Equation (6) is satisfied ($\omega_{D}$ = 1) [48,49].

(6) $$\omega_{D}=\int \frac{d{\bar{\varepsilon }}_{\mathrm{eq}}^{\mathrm{pl}}}{{\bar{\varepsilon }}_{0}^{\mathrm{pl}}\left(\eta ,{\overset{˚}{\bar{\varepsilon }}}_{0}^{\mathrm{pl}}\right)}=1$$

The state of stress at the point is defined in Equation (7) [28,48], where $\bar{\sigma }$ is the undamaged stress tensor:

(7) $$\sigma =\left(1-D\right)\bar{\sigma }$$

For the damage required to develop (${\bar{\varepsilon }}_{\mathrm{eq}}^{\mathrm{pl}}$ > ${\bar{\varepsilon }}_{0}^{\mathrm{pl}}$), the variable D increases from 0 to 1. At this maximum value, the material loses its load carrying capacity for the equivalent plastic strain (${\bar{\varepsilon }}_{\mathrm{eq}}^{\mathrm{pl}}$ = ${\bar{\varepsilon }}_{f}^{\mathrm{pl}}$). The damage development occurs when Equation (8) is satisfied [48,49].

(8) $$\omega_{D}=\int \frac{d{\bar{\varepsilon }}_{\mathrm{eq}}^{\mathrm{pl}}}{{\bar{\varepsilon }}_{0}^{\mathrm{pl}}\left(\eta ,{\overset{˚}{\bar{\varepsilon }}}_{0}^{\mathrm{pl}}\right)}\geq 0$$

The damage evolution is specified using fracture dissipation energy ($G_{f}$) before the damage initiation and equivalent plastic displacement at failure (${\overset{¯}{u}}^{\mathrm{pl}}$) after damage initiation. The fracture energy is measured based on the stress–displacement outcomes, as seen in Equation (9) [48].

(9) $$G_{f}=\int_{{\bar{\varepsilon }}_{0}^{\mathrm{pl}}}^{{\bar{\varepsilon }}_{f}^{\mathrm{pl}}}L\sigma_{y}d{\bar{\varepsilon }}^{\mathrm{pl}}=\int_{0}^{{\bar{u}}_{f}^{\mathrm{pl}}}\sigma_{y}d{\bar{u}}^{\mathrm{pl}}$$

The damage evaluation law [48,49] can also be defined using displacement at failure in two cases: linear form and exponential form. The governing equation for equivalent plastic displacement is

(10) $$D=D\left({\overset{¯}{u}}^{\mathrm{pl}}\right)$$

In this study, linear form is used, as the damage variable evolution is expressed in Equation (11) [48], where ${\overset{¯}{u}}_{f}^{\mathrm{pl}}$ = the effective plastic displacement.

(11) $$\dot{D}=\frac{{\overset{˚}{\overset{¯}{u}}}^{\mathrm{pl}}}{{\overset{¯}{u}}_{f}^{\mathrm{pl}}}$$

The relationship between effective plastic displacement (${\overset{¯}{u}}_{f}^{\mathrm{pl}}$) and energy dissipation ($G_{f}$) is defined in Equation (12) [48], where $\sigma_{y0}$ = yield stress value after reaching failure criteria. All of the composite types used in the study stopped immediately after the fracture point. The displacement at failure (${\overset{¯}{u}}^{\mathrm{pl}}$) is maintained at 0.05.

(12) $${\overset{¯}{u}}_{f}^{\mathrm{pl}}=\frac{2G_{f}}{\sigma_{y0}}$$

### 3.4. Numerical Material Modeling

The experimental TT results from the author’s previous study [31] were used as input parameters for numerical analysis in this study. These experimental TTs were performed using Zwick Roell (Z020) tester. The extensometer was connected digitally using testXpert II software to record the composite’s SS curves. For each composite type, 15 samples were tested. The tests recorded data for applied force (N) to its corresponding nominal strain (%). The force–strain readings ranged from 1250 per 100% EP sample (lowest) to 4800 per 60% rCF/EP composite sample (highest) per test. Overall, an average of 236,000 readings were recorded from the experimental TT.

In the measured experimental TT values, the Young modules represent the slope of the elastic zone. The yield points represent the transition phase from elastic to plastic and, finally, the plastic zone ended by reaching the ultimate points. The samples broke immediately after achieving their ultimate points, and the process ended with a minor value drop in the end. Furthermore, the data were processed. The force was converted into engineering stress (MPa) using the cross-section values of each sample, and the strain was kept unitless. Out of the obtained SS curves, 15 curves (minimum) per composite type—EP, (40 and 60%) rGF/EP and rCF/EP, a single SS curve per composite type has to be chosen instead of performing an average to combine the 15 curves per sample. One best sample per composite type, with the SS curve occupying the average position from the overall population, was selected. By doing so, real-time measured composite behaviours were preserved compared to averaging the SS values to obtain one unified curve (totally modified SS values).

After selecting five SS curves (one per composite type), standard formulas were used to convert them into true SS values. During the experimental TT, samples were held using pneumatic holders, causing the samples to experience micro compression. The sensitive extensometer also recorded such behaviours, clearly visible in the measured data as negative values. Hence, these values below zero were eliminated from the true SS data. Then, these data were further processed using Abaqus inbuilt material calibration tool. The true SS values were fed as data sets (input). A SS curve appears based on the input with strain as *x*-axis and stress as *y*-axis. Before initiating the actual calibration, curve smoothing was performed with 0.75 weight. Next, an elastoplastic isotropic behaviour was created to calibrate the inputs. A manually operated pendulum impact tester was used to perform the experimental charpy unnotched IT. Similar to the TT, 15 samples (minimum) per composite type were tested. However, only the TT data were further processed and used as an input for the simulation.

### 3.5. Modelling, Loads and Boundary Conditions

#### 3.5.1. Tensile Test Model

**Figure 5:**
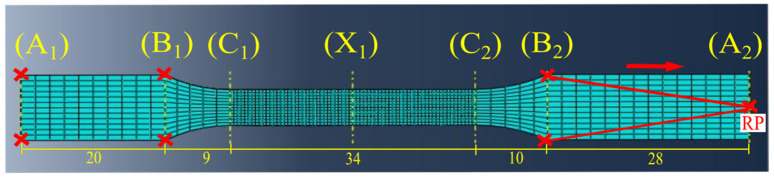
The modelled TT sample with boundary conditions and loads.

The numerical TT simulation was performed using a dogbone-shaped sample. The sample was designed and extruded based on ISO 527-2 specimen type 1BA standard [32], similar to the samples used during the experimental TT. After the primary modelling, based on the overall sample length of 100 mm, it was divided into seven points, which were A_1_, B_1_, C_1_, X_1_, C_2_, B_2_ and A_2_, and five cells, which were A_1_–B_1_, B_1_–C_1_, C_1_–C_2_, C_2_–B_2_ and B_2_–A_2_, in that order (see Figure 5).

The sample meshing was performed at each cell based on the created seven datum planes on each point, perpendicular to the sample length. A fine mesh was created between points A_1_ to C_1_ and C_2_ to A_2_ with 4080 elements. These are the cells experiencing the least stress. The maximum stress will be experienced within the cell C_1_–C_2_, leading to damage at X_1_ (expected point). A very fine mesh was created with 8160 elements, covering the sensitive area. An explicit mesh with linear geometric order was used, with full integration eliminating the hourglass effect throughout the sample.

The dogbone sample was fixed at the bottom cell A_1_–B_1_, restricting free movements in terms of displacement (U) and rotation (UR) in all three (x,y,z) axes (U1 = U2 = U3 = UR1 = UR2 = UR3 = 0). Similar restrictions were applied to cell B_2_–A_2_ at the opposite end (U1 = U3 = UR1 = UR2 = UR3 = 0), but allowing displacement (U2) in the direction parallel to the applied force (*y*-axis). This particular condition was assigned to the sample via a separately created reference point (RP) at point A_2_. The cells occupying the space between points B_2_ and A_1_ were couples to the created RP. During the numerical TT simulation, the applied displacement (U2) will pull the cells uniaxially at RP and evenly distribute the force within the respective cells below.

#### 3.5.2. Impact Test Model

**Figure 6:**
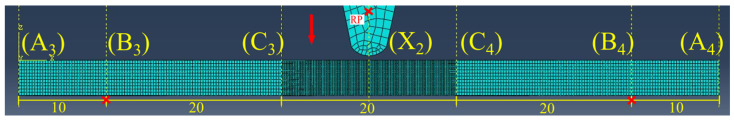
The modelled IT sample with boundary conditions and loads.

The numerical IT was performed using a rectangular sample and an impactor (hammer). The sample was designed and extruded based on the dimensions from ISO 179-1 type 1 test specimens 80 mm × 10 mm × 4 mm under charpy unnotched impact strength [33]. The actual dimensions of the hammer were measured and modelled to achieve a closer simulation to experimental IT. After the primary modelling, based on the overall sample length of 80 mm, it was divided into seven points, which were A_3_, B_3_, C_3_, X_2_, C_4_, B_4_ and A_4,_ and five cells, which were A_3_–B_3_, B_3_–C_3_, C_3_–C_4_, C_4_–B_4_ and B_4_–A_4_, in that order (see Figure 6). The parts (sample and hammer) were then assembled by placing the hammer tip above X_2_, maintaining a small gap between the parts.

The sample meshing was performed at each cell based on the created seven datum planes on each point, perpendicular to the sample length. A fine mesh with 63,800 elements was created between points A_3_ to C_3_ and C_4_ to A_4_. The points cover four cells that were not exposed directly to impact, making it a less sensitive area. The hammer hits perpendicular to the middle cell at point X_2_. This sensitive region of the sample experiences heavy damage, leading to a break. A very fine mesh with 63,800 elements was created at the middle cell C_3_–C_4_. An explicit mesh with linear geometric order was used throughout the sample. Full integration was used at the impact cell C_3_–C_4_, but reduced integrations were adopted at cells with no direct contact with the hammer in order to reduce the complexity. The hourglass was blocked from the entire model.

The meshed sample was pinned parallel to the impact surface at points B_3_ and B_4_. The displacement in all three (x,y,z) axes (U1 = U2 = U3 = 0) was restricted at the pinned edges. The hammer was defined as a rigid body by creating constraints. A normal mesh with 1786 elements was created for the hammer. A reference point (RP) was created at the centre of the hammer to apply uniform velocity perpendicular to the sample. Furthermore, contact modelling was implemented. The hammer (moving part) was adjusted until its surface fully covered the sample’s surface (inert part) during impact. The datum planes were used as a guideline to align the parts. After fixing the positions, surfaces were created using designated cells. An explicit contact-based interaction was created to assign the surfaces. First, a frictionless contact was created on the surfaces. Then, for the numerical IT, a tangential behaviour was created on the surfaces, with a 0.3 friction coefficient, followed by creating a normal behaviour with hard contact for the hammer to hit the sample, due to the applied velocity.

## 4. Results and Discussion

### 4.1. Numerical Input Parameters

Table 2 presents the input parameters for the numerical simulation. The experimental TT results were calibrated as elastoplastic isotropic behaviour to model the non-linear behaviour of the recycled composites numerically. These calibrated values are primarily used as input parameters to perform numerical TT and IT. The elastic region (linear) was defined using the Young modulus and Poisson ratio. The plastic region (non-linear) was defined using values between yield stress and ultimate points. Furthermore, these obtained a plastic stress (yield stress), and the plastic strain values were processed by excluding the SS values below the yield point, as the values were already defined as the elastic region. The damage initiation and development occurs after the ultimate points.

Therefore, damage prediction was included using non-calibrated parameters obtained from experimental TT results, such as fracture strain, stress triaxiality, strain rate and displacement at failure. These parameters were recorded during the experimental TT and further incorporated as input values for modelling. The stress triaxiality was −0.333 for all of the models. The strain rate was maintained as zero for the TT and as one for the IT. During TT, the displacement at failure for the rCF/EP and rGF/EP samples was maintained as low, in a range between 0.03–0.06, as the applied tension was parallel to the fibre direction. The displacement was raised to 0.15–0.17 for all of the composite types during IT, including EP during its TT (contains no fibre), as the damage occurred immediately at the composite’s ultimate point. The calibrated values recorded after were removed, as the values displayed a minor drop (neglectable), indicating that the recycled composite samples experienced a break, and no further significant SS values were recorded.

### 4.2. Numerical Tensile Test Results

**Figure 7:**
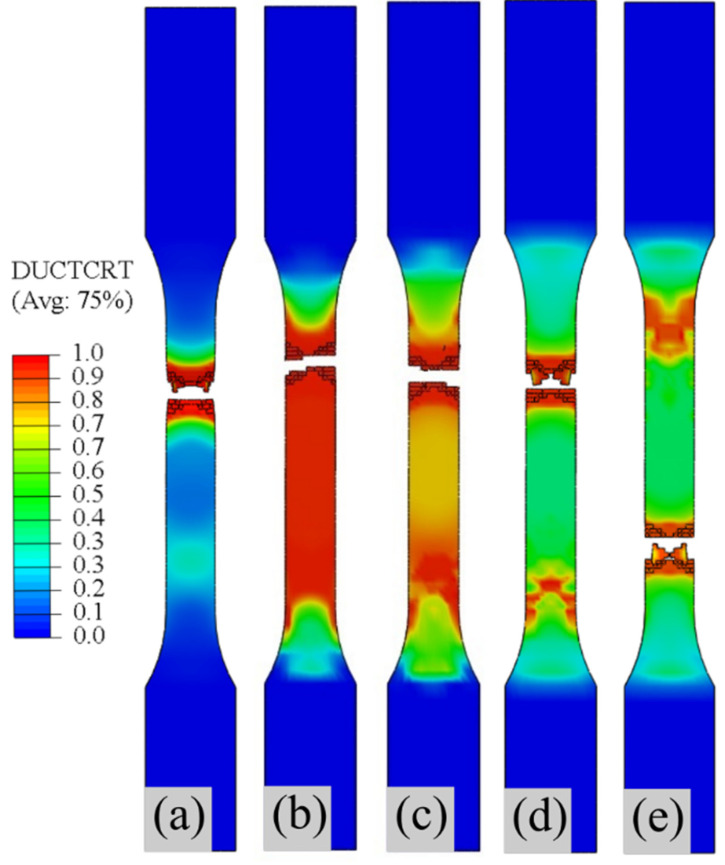
Fractured samples after numerical TT: (**a**) 100% EP; (**b**) 40% rGF/EP; (**c**) 60% rGF/EP; (**d**) 40% rCF/EP; (**e**) 60% rCF/EP.

Figure 7 presents the fractured samples of all composite types after numerical TT. The sample damage occurred based on ductile damage modelling (DUCTCRT). As can be seen, the ductile damage criteria distribution diversified across the composite types. As the variable D increases from zero to one, the materials lose their load carrying capacity for the equivalent plastic strain. At this phase, the progress in break initiation and development appears across the samples, depicting the overall fracture. The samples were fractured based on the applied tensile displacement at the sample’s reference point (RP) A_2_ (see Figure 5). As the tensile strength of the rGF/EP and rCF/EP samples increased, the displacement to failure also increased, with an increase in strain rate to failure. Initially, multiple estimations were made to fracture the samples within the defined time step. A lower displacement failed to damage the samples, and higher values exceeded the experimental time frame, resulting in errors. Finally, the optimal values of the applied displacement are as follows: 0.55 mm (40% rGF/EP), 0.67 mm (60% rGF/EP), 1.05 mm (40% rCF/EP), 1.15 mm (60% rCF/EP) and 2 mm (EP). It can be observed that the fibreless EP samples required a higher displacement to failure, indicating the longer strain rate to failure.

As expected, the numerically fractured samples appear to have similarities compared to their experimentally fractured samples, shown in Figure 3. In the EP samples, the fibreless material showed minor necking at the fractured region. Such behaviour was not visible in the experimental TT sample, despite the EP samples being brittle. In the rGF/EP samples, the damage initiation and development were evenly spread throughout the sample’s midsection (see Figure 7b,c), unlike other composite types (EP and rCF/EP), with damage behaviour in a particular spot. Such behaviour in the rGF/EP samples could be due to the thoroughly wet, long and UD rGFs evenly distributing the applied tension. However, as the rGFs volume increased (40% to 60%), the samples gradually transformed towards similar damage behaviour, such as EP and rCF/EP. In the rCF/EP samples, the resin-rich zones, which originated due to the poorly wet rCFs clusters, weakened the samples in uneven spots and created damage initiation in more than one spot at the sample’s midsection (see Figure 7d,e). The numerical simulations assume the composites are defect-free, as no external errors are modelled, yet the input parameters are derived from the experimental results. The minor defects reflected within the experimental results are also visible to a certain extent in the numerical TT results.

Furthermore, the numerical tensile results of all of the composite types were analysed. The results from the FE models were recorded as frequencies, with 200 intervals recorded from the start to the end of the experiment based on evenly spaced time intervals. The recorded results for each composite type were extracted using the ODB field output. As the samples experienced maximum stress at the fractured area, one element was picked to plot their respective values. Finally, the maximum principal stress (*y*-axis) and respective logarithmic strain (*x*-axis) for all of the composite types at the damaged region were plotted and compared to their respective experimental tensile SS values.

**Figure 8:**
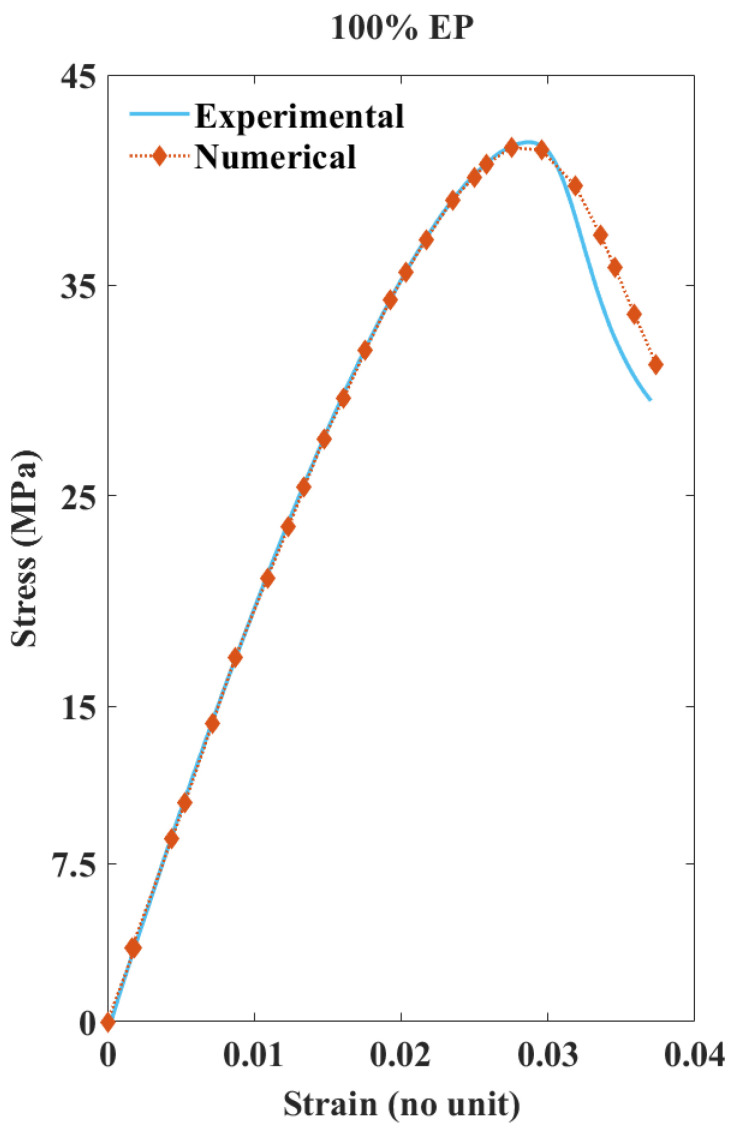
Comparing experimental and numerical SS curves of 100% EP composite.

Figure 8 presents the plotted SS values of experimental vs. numerical for the 100% EP composite sample. The experimental TT results (15 samples minimum) displayed a 2.69 coefficient of variation (CoV) for the tensile strength and 2.09 CoV for the tensile modulus; the SS curves in particular displayed uniform consistency. These results make EP a highly reliable composite type to validate the adopted numerical method. Typically, failure initiation for fibreless polymers under tension occurs as the strain rate increases, decreasing its overall stiffness [52]. Similarly, EP samples fractured at a higher strain among all of the tested FRP composite types. The numerically extracted SS values from the FE models were further processed by eliminating additional values after the fracture region. As can be seen, the numerical SS values fit both the linear and non-linear paths of the experimental SS values. The predicted damage values from the fracture initiation to development slightly deviate from the actual damage values. However, they lie within an acceptable range. From the results, it can be concluded that the adopted FE methodology is capable of recording the TT results numerically for highly consistent material.

**Figure 9:**
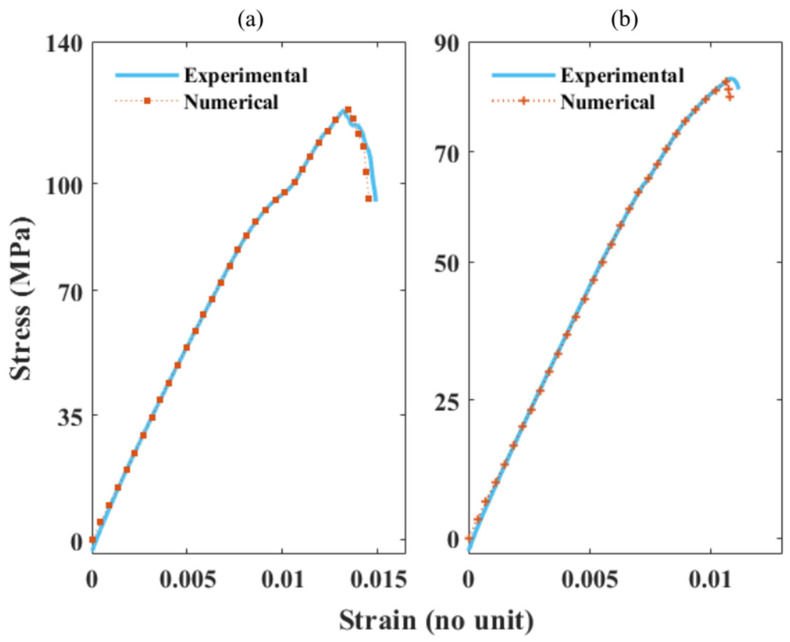
Comparing the experimental and numerical SS curves of rGF/EP composites: (**a**) SS curves of 60% rGF/EP; (**b**) SS curves of 40% rGF/EP.

**Figure 10:**
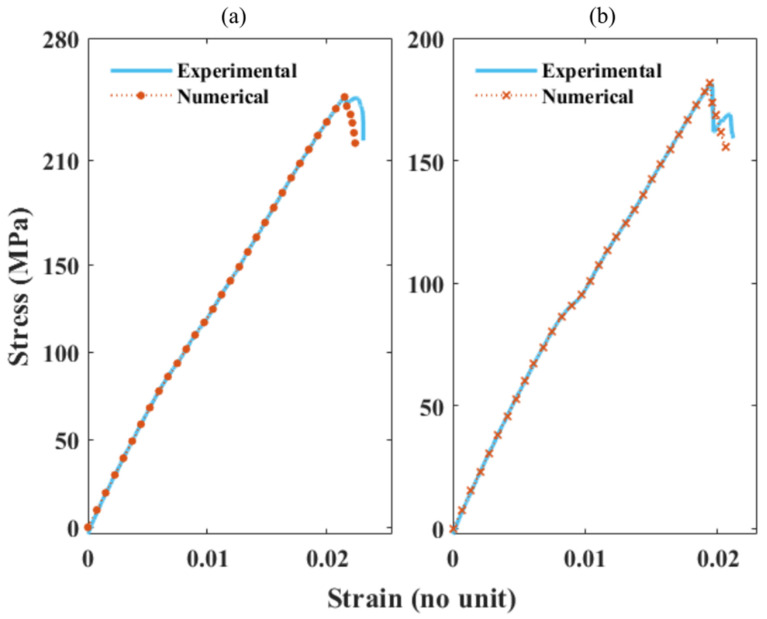
Comparing experimental and numerical SS curves of rCF/EP composites: (**a**) SS curves of 60% rCF/EP; (**b**) SS curves of 40% rCF/EP.

Figure 9a,b presents the plotted experimental vs. numerical SS values for the 60 and 40% rGF/EP composite types. The composites possess a lower strain rate compared to the EP sample results. As the fibre volume increases, the strain rate decreases, resulting in a higher stress value. Figure 10a,b presents the plotted experimental vs. numerical SS values for the 60 and 40% rCF/EP composite types. In vCF/EP, composites containing a higher fibre volume (60%) exhibit a reduced inter-fibre distance. This increases the residual stress, causing the damage initiation at lower SS rates [42]. In addition, UD vCF/EP laminate with resin-rich spots fail at a lower strain rate and possess a lower stiffness, despite holding an average fibre volume [27]. Similarly, the poorly wet (40 and 60% rCF/EP) and higher fibre volume (60% rCF/EP) composite samples displayed damage initiation and development at a high stress rate, with a relative lower strain rate ratio when compared to EP and rGF/EP.

As seen in Figure 9 and Figure 10, the numerical SS values fit the non-linear paths of the experimental SS values. However, the predicted damage values did not accurately follow the path of the experimental values. Regardless, they lie within the range. Such deviations are expected, as the rCF/EP and rGF/EP are complex materials. The numerical modelling did not incorporate the external defects, such as the resin-rich zone and unwet fibres in rCF/EP samples and char formation in rGF/EP samples, that significantly influenced the sample fracture behaviour during experimental testing. Alternatively, the FE models predicted a defect-free damage behaviour. In 40 and 60% rGF/EP, the predicted damage coincides with a higher accuracy than the rCF/EP samples. The damage predictions seem to be dropping directly from their ultimate points in (40 and 60%) rCF/EP and 60% rGF/EP, instead of following the non-linear experimental damage range.

**Figure 11:**
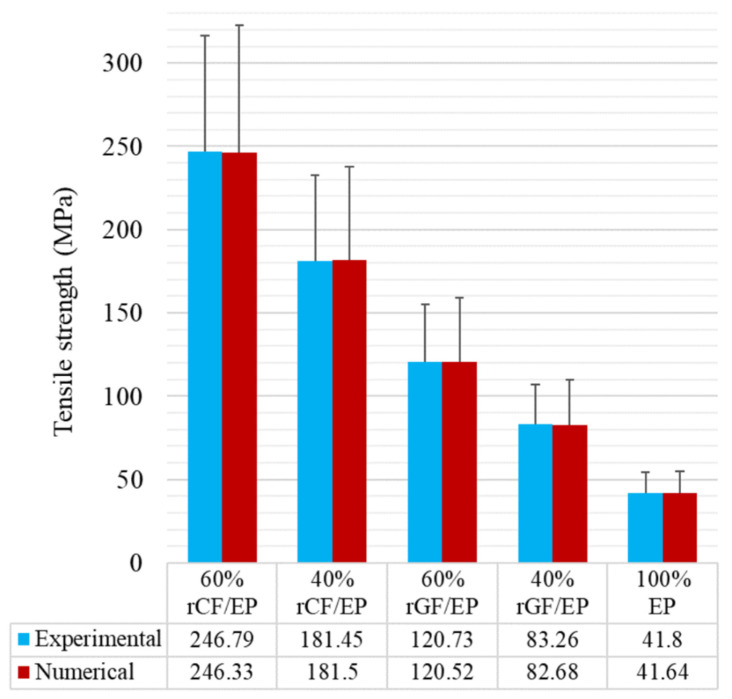
Measured tensile strength of the composites experimental vs. numerical.

Figure 11 presents the overall TT results of experimental and numerical analyses for all of the composite types. The tabulated values were obtained from the true stresses. As can be seen, the numerically measured tensile strength appears to be similar when compared to the experimental values. Furthermore, standard deviation (SD) values were calculated from stresses of each composite type and incorporated as error bars. The SD of experimentally measured stresses and numerically obtained maximum principal stresses were compared. Despite the numerical SS, values were measured at particular intervals in order to reduce the computing complexity. The values seem within the range of higher-order SS values from the experimental test. Based on the results, it can be concluded that the adopted FE methodology is capable of recording the TT results numerically for inconsistent materials.

### 4.3. Numerical Impact Test Results

**Figure 12:**
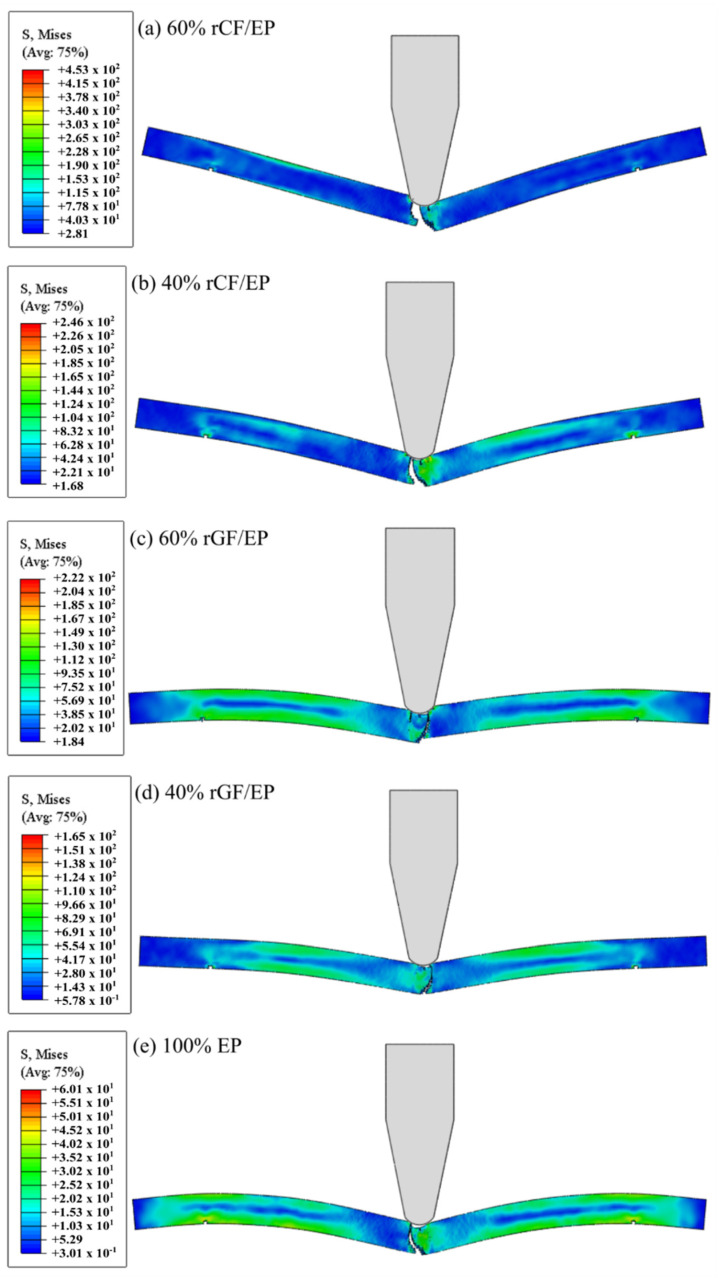
Numerical IT results using von Mises stress distribution: (**a**) 60% rCF/EP; (**b**) 40% rCF/EP; (**c**) 60% rGF/EP; (**d**) 40% rGF/EP; (**e**) 100% EP.

Figure 12 presents the von Mises stress distribution across the numerical IT samples. As the hammer impacted the sample, variable D increased from zero to one, causing crack initiation and development at the hammer’s contact point with the sample. Typically, von Mises stresses are used to estimate yield failures in ductile materials. As the ductile damage model (DUCTCRT) was used to study the recycled composites, the von Mises stress distribution projects an understanding of the plasticity for all of the tested composite materials. As seen from all of the composite types, the majority of the elements experienced mid-range stress. However, the scattered elements at critical points, such as pinned regions and inner fractured regions, reached the maximum stress value. The test was performed until the samples experienced a complete fracture. The samples displayed an exponential decrease in their overall stiffness in the following order: 60% rCF/EP (stiffest), 40% rCF/EP, 60% rGF/EP, 40% rGF/EP and EP (high-plasticity). The EP samples appear more highly flexible than fibre-reinforced composites (rGF/EP and rCF/EP), representing the absence of fibre (low-elasticity). Overall, as the fibre volume and the composite strength increased, the Young modulus increased, resulting in a stiffer composite.

In the impact mode arrangement, the direction of applied force was perpendicular to the recycled fibre direction, similar to a three-point bending test. As seen in Figure 12, the composite types 40% rCF/EP and 60% rGF/EP experienced maximum stresses that resembled each other. However, the stress concentration for 60% rCF/EP was higher when compared to the rest of the composite types, especially 40% rCF/EP. The 20% increase in the fibre volume fraction increased the stress concentration by 84.14%, making it a robust composite in an impact mode. However, the 20% increase in the fibre volume for rGF/EP resulted in a 34.54% increase in the von Mises stress. As expected, the homogeneous EP samples failed, encountering a 63–84% lower stress range compared to the rCF/EP and rGF/EP composite types. However, it possessed a high stress distribution across the sample.

**Figure 13:**
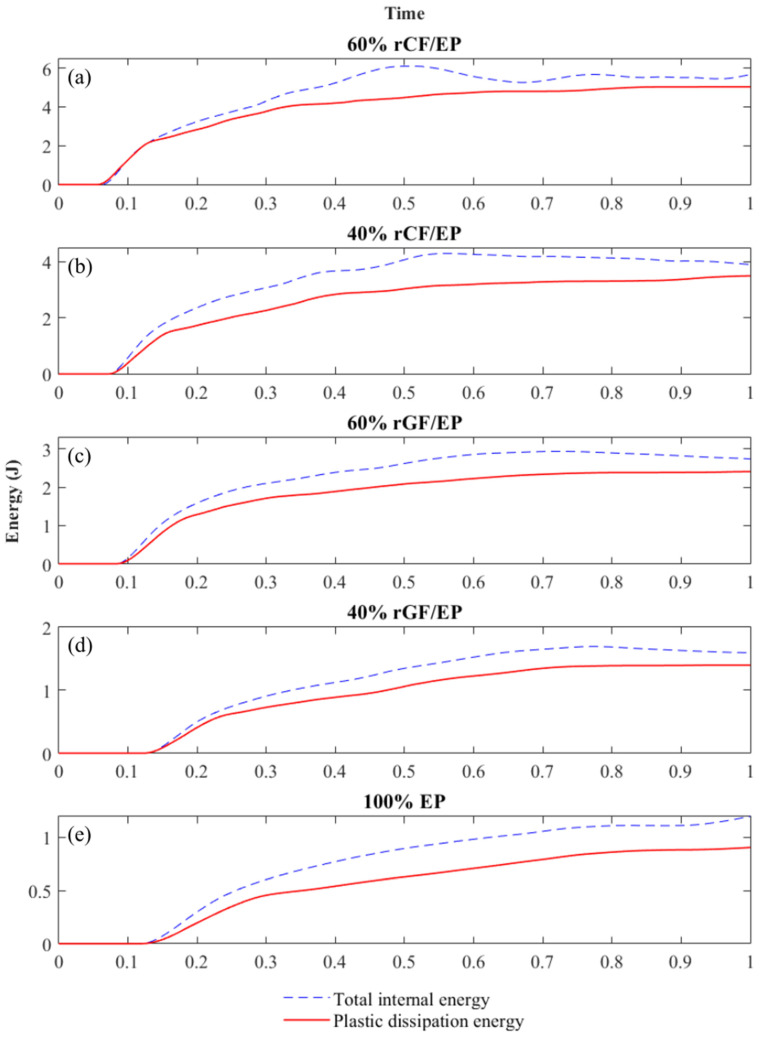
Internal energy observed by the composites after numerical IT: (**a**) 60% rCF/EP; (**b**) 40% rCF/EP; (**c**) 60% rGF/EP; (**d**) 40% rGF/EP; (**e**) 100% EP.

Figure 13 presents the total internal energy and plastic dissipation energy observed by the composite samples after the numerical IT. The energy measurement starts as the hammer contacts the sample (damage initiation). Further, as the hammer progressed based on the applied velocity and time step, the crack propagation parallel to the impact direction finally ends with a fracture. Thus, the recorded maximum energy at fracture was taken as the plastic dissipation energy observed by the samples. During numerical IT, the energy dissipation in the samples increases with respect to the hammer velocity, and is required to establish a boundary [53]. Such entities were also observed during the simulation process. The modelling failed with errors at higher velocities, as the sample energies were not fully recorded based on the established time interval, and at lower velocities, the samples remained undamaged. Finally, after repeated estimations using lower velocities, the hammer velocity for all of the composite types were fixed as follows: 8.1 m/s (60% rCF/EP), 7.1 m/s (40% rCF/EP), 6 m/s (60% rGF/EP), 4 m/s (40% rGF/EP) and 4.25 m/s (EP).

The model outcomes were susceptible to three significant parameters—the mesh size, applied velocity (hammer velocity) and time interval (step)—for the experiment to occur. Even minor modifications in these parameters influenced the overall energy values. After various combinations of remodelling the parameters to obtain results closer to the actual events, two parameters—time interval and mesh size—were fixed for all of the composite samples. The impact velocity of the hammer varied for each composite type, exponentially increasing from 40% rGF/EP, 100% EP, 60% rGF/EP and (40% and 60%) rCF/EP, in that order. In addition, during experimental IT, the hammer size increased exponentially as the fibre volume and the composite strength increased.

**Figure 14:**
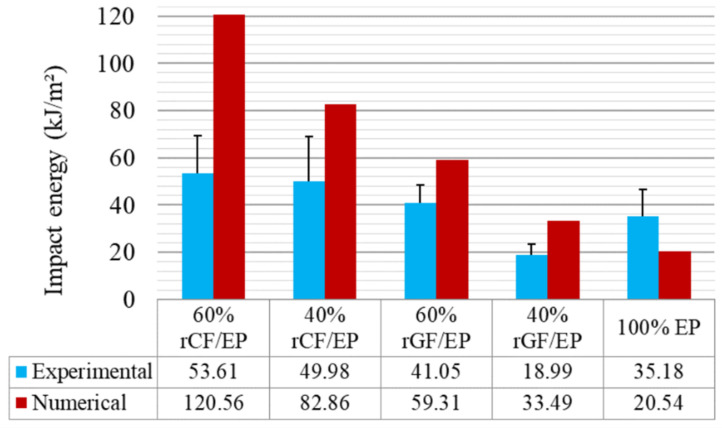
Measured impact strength of the composites experimental vs. numerical.

Figure 14 presents the impact energy results from experimental and numerical analyses for all of the composite types. The SD values from the experimental IT of each composite type—15 samples minimum per composite type—were incorporated as error bars. The numerical IT simulations are unified to eliminate the model’s errors, and one test per material was performed, leaving no room for SD. As seen, the numerical results seem to be higher than the experimental results. As the fibre volume and sample strength decreased, the impact resistance of the samples also decreased gradually. The rCF/EP sample displays a high variation compared to other composite types. Such variation could be due to the local defects (poorly wet rCFs and high fibre volume) involved in the experimental IT samples. They possess a higher CoV between the sample population: 29.62 CoV for 60% rCF/EP and 38.40 CoV for 40% rCF/EP sample types. Meanwhile, the numerical IT was predicted based on the elastoplastic behaviour, in which, external defects were absent.

Similarly, the experimental IT rGF/EP samples, despite possessing a comparatively lower CoV of 18.48 for the 60% rGF/EP and 24.20 for the 40% rGF/EP sample types, contained random char distribution (local defects) across the sample. This significantly influenced fracture behaviour. Hence, the predicted IT also neglected such defects, exhibiting the impact results for defectless rGF/EP samples. In contrast, EP samples displayed explosive breaking during experimental IT due to their high hardness property, whereas the FE model tested the samples step-by-step, avoiding such explosive behaviour and recorded a lower impact behaviour than experimental IT.

The energy difference within the 40% rGF/EP and 60% rGF/EP composite types in the experimental case is 22 kJ/m^2^, and the numerical case is 25 kJ/m^2^. However, the difference between 40 and 60% rCF/EP in the experimental case is 3.63 kJ/m^2^, and in the numerical case is 37.7 kJ/m^2^. Such a poor energy difference between 40 and 60% rCF/EP experimental IT can be related to previously mentioned defects, such as the poor wettability of rCF to reinforce with EP, as the energy pattern in other composite types seems to fall within a comparable order. However, the char-based defects on rGF/EP were reflected less. Overall, based on the energy variations in rGF/EP and rCF/EP, it can be concluded that the numerically observed energies are reliable in all composite types, and are defect-free.

Furthermore, when comparing the numerical IT results to literature-based experimental IT studies, the energy absorbed by 40% rCF/EP is 82.86 kJ/m^2^, and by 60% rCF/EP is 120.46 kJ/m^2^. Meanwhile, Caminero et al. 2016 [54] tested UD 66% vCF/EP using unnotched charpy IT and noticed a 189.01 kJ/m^2^ internal energy. The study highlighted that, among various multidirectional vCF/EP laminates, the UD laminate possesses a high performance under impact and flexural testing. In addition, when comparing the energy absorbed by 40% rGF/EP, which is 1.4 kJ or 33.49 kJ/m^2^, and 60% rGF/EP, which is 2.4 kJ or 59.31 kJ/m^2^, to a similar study, Bazli et al. 2019 [55] tested UD 70.5% vGF/EP composites under unnotched charpy IT, which resulted in 5.6–7.1 kJ depending on the exposed temperature. The study also highlighted that UD vGF/EP displayed a higher performance in flexural and impact modes compared to a woven and randomly oriented fibre arrangement.

It can be noted that the numerical predicted IT results of the recycled composites are not identical when compared to literature studies with their virgin counterpart. Regardless, they are higher than their experimental IT results. This could be due to the lack of input parameters concerning the composite’s shear properties. The impact direction is perpendicular to the fibre orientation, demanding interlaminar shear characters for higher-order modelling. In addition, the numerical IT model’s sensitive status towards the mesh size, hammer velocity and time interval also significantly influence the results.

## 5. Conclusions

In this study, the recycled composites (40 and 60%) rCF/EP and rGF/EP, along with cured EP samples, were numerically investigated using FE methods. The study primarily investigated the uniaxial tensile and impact resistance properties of the recycled composites. The FE modellings were developed based on the elastoplastic behaviour and ductile damage failure. The experimentally measured uniaxial tensile results from the previous study [31] were utilised as input parameters for modelling. All of the composite types—EP, rCF/EP and rGF/EP—were successfully fractured using the developed models in tensile and impact loading conditions.

The FE models managed to record the non-linear behaviours of the recycled composites from the numerical TT. Simultaneously, it predicted the recycled composite’s damage initiation and development under tensile loading. The numerical measured maximum principal stress and logarithmic strain from the dogbone-shaped samples showed that the applied displacement to failure increases as the overall strain rate increases. The fibreless EP samples possessed a higher displacement to failure (2 mm), which explains their higher strain rate and associated plasticity—followed by 60 and 40% rCF/EP and rGF/EP. The numerical results mapped the non-linear behaviour of the composites until damage initiation with a higher accuracy. However, the predicted damage behaviour did not precisely replicate the damage path. Regardless, they lie within an acceptable range.

The results from the contact-based FE models under impact loading conditions with rectangular samples and a hammer (impacter) were investigated using the von Mises stress distribution. The results showed that, as the fibre volume and the composite strength increased, the composites became stiffer, with the 60% rCF/EP sample being the stiffest and fibreless EP samples exhibiting a high plasticity. The 40% rCF/EP and 60% rGF/EP exhibited similar stress concentrations. Similarly, the impact velocity increased as the composite strength increased, making 60% rCF/EP samples the toughest and 40% rGF/EP the weakest to impact resistance. Furthermore, the damage behaviours for impact loading were investigated by the energy observations. The total internal energy and plastic dissipation energy were measured from the FE models. These observed energies were higher than their experimental impact energies, but lower than similar virgin composites from the literature. Such results were expected, as the FE models did not include any external defects from the recycling process that influenced the experimental results significantly, such as a poor resin wettability in the 40 and 60% rCF/EP samples, a bundle formation in high fibre volume fraction in the 60% rCF/EP samples and a char formation in the rGF/EP samples. Alternatively, the FE models predicted defect-free damage behaviours and energy absorption for the recycled composite.

The numerical IT was highly sensitive to three primary numerical parameters: sample mesh size, hammer velocity and experimental time (step time). A trivial change in these parameters will drastically influence the total internal energy absorbed by the samples. However, by fixing the mesh size and time step as constant and modifying the impact velocity based on the sample type, such defects can be overcome.

It is concluded that the FE methodology developed to numerically investigate the mechanical properties (tensile and impact) and predict the damage behaviour of rCF/EP and rGF/EP composites have shown substantial results. Hence, the adopted modelling technique can validate experimental results of recycled composites possessing complex and inconsistent mechanical behaviours. The study will provide insight towards investigating recycled composites containing defects from recycling methods numerically. Further improvements are required to overcome certain limitations. For meso-scale modelling, the recycled composite’s material properties in compression, flexural and shear are required to obtain input parameters to define the elastic region of the fibres. By doing so, the composites can be defined as transversely isotropic and even anisotropic. For micro-scale modelling, the individual recycled fibre’s tensile properties are required to define the FRP composites as heterogeneous solids in order to analyse the fracture mechanics under various loading conditions. The future research directions are aimed to optimise such limitations in order to map the mechanical properties and fracture behaviours of the recycled FRP composites closer to real-time.

## Figures and Tables

**Table 1:** Experimental results of the compression moulded recycled composites [31].

Composite RECIPES	V^f^ (wt%)	V^r^ (wt%)	Tensile Strength (MPa)	Young Modulus (GPa)	Impact Strength (kJ/m^2^)	Fracture Strain (No Unit)	Density (g/cm^3^)
rCF/EP	60 ± 2	40 ± 2	235.70	60.80	53.61	0.00683	1.52
	40 ± 2	60 ± 2	210.34	45.28	49.98	0.00827	1.64
rGF/EP	60 ± 2	40 ± 2	114.58	30.72	41.05	0.00272	1.77
	40 ± 2	60 ± 2	65.42	27.37	18.99	0.00156	1.85
EP	0	100	39.46	2.16	35.18	0.05810	1.45

**Table 2:** Primary input parameters for numerical testing.

Composite Types	Young Modulus (MPa)	Yield Point		Ultimate Point		Fracture Strain	Poisson Ratio
		Stress (MPa)	Strain (No Unit)	Stress (MPa)	Strain (No Unit)		
60% rCF	13,262	55.8332	0.00421	246.529	0.02151	0.00683	0.3
40% rCF	11,103	45.8554	0.00413	181.254	0.01952	0.00827	0.3
60% rGF	10,921.3	26.4294	0.00242	120.598	0.01321	0.00272	0.25
40% rGF	9011.50	22.0782	0.00245	83.16	0.01077	0.00156	0.25
100% EP	2004.46	10.1626	0.00507	41.7633	0.02825	0.05810	0.3

## Data Availability

The data presented in this study are available on request from the corresponding author.
